# Association between toll-like receptors 9 (TLR9) gene polymorphism and risk of pulmonary tuberculosis: meta-analysis

**DOI:** 10.1186/s12890-015-0049-4

**Published:** 2015-05-08

**Authors:** Zhi Chen, Wei Wang, Jianqin Liang, Jinhe Wang, Shisheng Feng, Guangyu Zhang

**Affiliations:** Department of Tuberculosis, The 309th hospital of PLA, No. 17, Heishanhu Road, Haidian District Beijing, 100091 China

**Keywords:** Toll-like receptor 9, Polymorphism, Tuberculosis, Meta-analysis

## Abstract

**Background:**

Previous studies indicated that the single nucleotide polymorphisms (SNPs) in TLR9 gene might be associated with Tuberculosis (TB) risk. However, the results are inconsistent and inconclusive.

**Methods:**

1745 articles from four databases were involved in our study. A meta-analysis on the associations between the seven polymorphisms and TB risk was carried out by comparison using different genetic models.

**Results:**

In this systematic review 8 studies from seven English articles were analyzed. Our results showed that rs352139 is significantly associated with TB risk (AA vs. AG, OR 0.77, 95% CI 0.65-0.92, P = 0.004). In the ethnic subgroup analysis, Indonesians with AA genotype had a decreased susceptibility while Mexicans with GG allele had an increased risk.

**Conclusions:**

The meta-analysis indicated that rs352139 polymorphism might be associated with decreased TB risk in Indonesians whereas increased risk in Mexicans. Whether the observed association was due to causal effect needs to be further studied.

## Background

Pulmonary tuberculosis (TB) caused by *Mycobacterium tuberculosis* (MTB), is one of the most contagious diseases in humans. A recent report (2014) from WHO estimated that one third of the global population has been latently infected without clinical symptoms. In 2012, approximately 8.6 million people worldwide were diagnosed with TB and 1.3 million died from TB [1]. MTB could infect human by invading antigen presenting cell (Macrophages and Dendritic cells) in the lung. But only 5-10% of infected people developed clinical symptoms probably due to undermined immunity response [1].

Toll-like receptors (TLRs) are pattern recognizing receptors (PRRs) and play an important role in regulating human’s immune system. The TLRs can recognize pathogen-associated molecular patterns (PAMPs) in both extracellular and intracellular environment. The TLRs have the ability to initiate signaling pathways that are responsible for activating both innate and acquired immune responses as well as the production of inflammatory cytokines. The members of TLRs family have been detected on human plasma membrane (TLR1, TLR2, TLR4-6) and in leukocyte endosome (TLR3, TLR7-9) [2]. For the risk of MTB infection, the most important TLRs are TLR1, TLR2, TLR4, TLR6 and TLR9 [3]. TLR1 combines with TLR2 as a heterodimer and recognizes triacylated lipopeptides of MTB. TLR2-TLR6 complex recognizes diacylated lipopeptides and bacterial LTA of Mycoplasmal compounds. TLR4 recognizes lipopolysaccharide (LPS) and TLR9 homodimer recognizes the exogenous MTB DNA as PAMPs [4]. Studies focusing on TLR1, 2, 4 and 6 have found that single nucleotide polymorphisms (SNPs) of these TLRs genes might be associated with differential TB risk. However, neither systematic review nor meta-analysis has been reported for TLR9 [3].

The gene of toll-like receptor 9 is located on chromosome 3p21.3. The total length of TLR9 gene is approximate 5 kb. Its coding gene has two exons, and the major coding region is in the second exon [5]. Based on NCBI SNP database, twelve SNPs have been identified for TLR9 gene, in which NM_017442.3:c.-1486 T > C (rs187084) located at the upstream of promoter may be an important one [6]. Previous study it has shown that C genotype was associated with reduced TLR9 transcription activity when compared with T genotype [7], indicating population with C genotype may be susceptible to diseases related to TLR9 gene.

Researches have indicated certain race population with special genotype of TLR9 polymorphism might have higher risk for TB; however the findings are inconsistent and inconclusive. From public health perspective, given the widespread and severe nature of the disease, it is important to understand the association between TLR9 polymorphism and TB risk, so that individuals with higher risk genotype could be identified and receive targeted preventive care. In this study, we conducted a systematic review of current literature on this issue and analysis of the associations between TLR9 polymorphisms and TB risk.

## Methods

### Literature review

**Figure 1:**
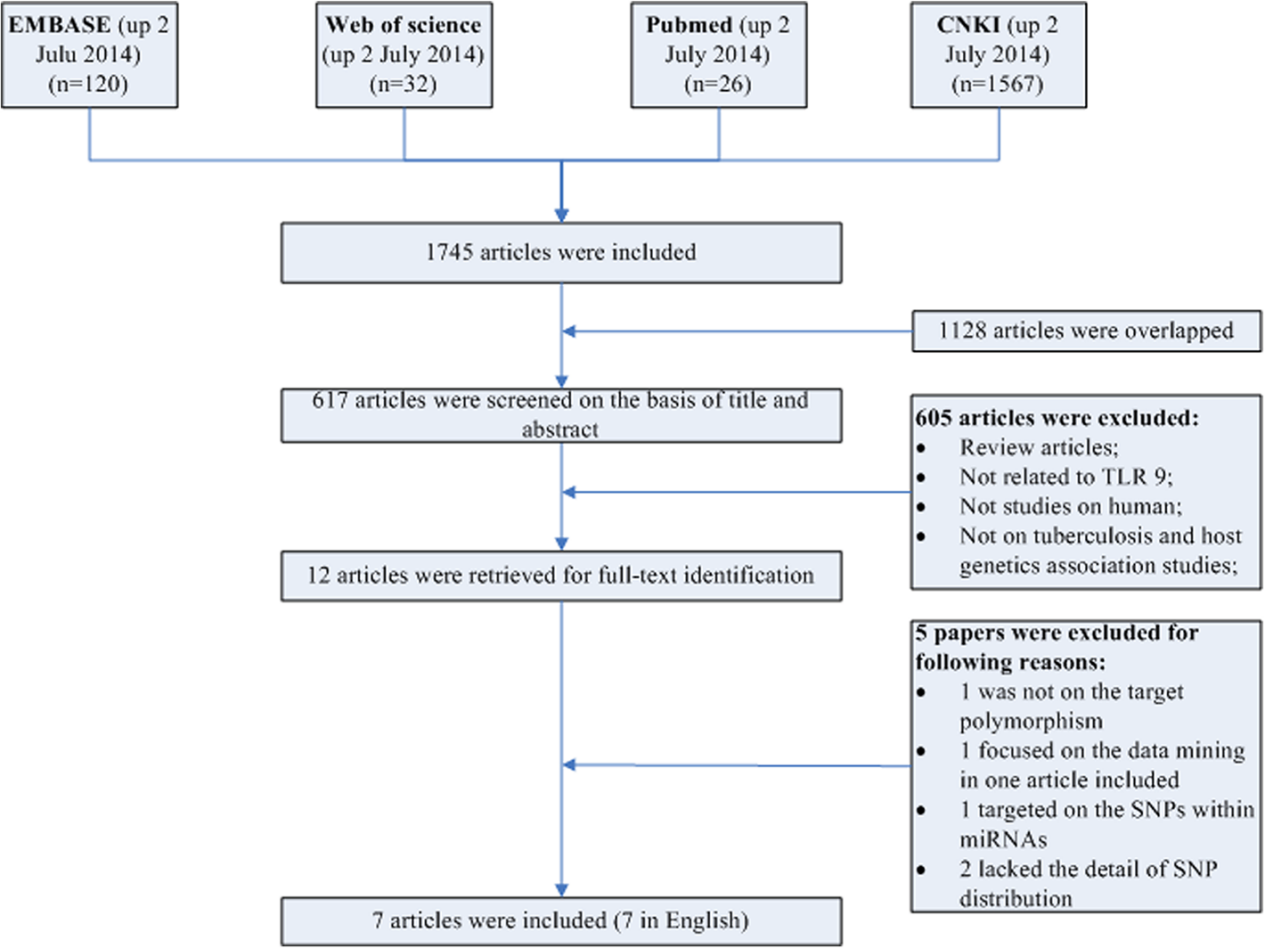
Flow chart showing the study selection procedure. CNKI: China National Knowledge Infrastructure.

We searched the EMBASE, Web of science, PubMed and Chinese National Knowledge Infrastructure (CNKI) databases to identify the publications that reported the association between the TLR9 polymorphism and risk for pulmonary tuberculosis from April, 2004 to July, 2014. The key words used were ‘toll like receptor 9, SNP, lung tuberculosis’, ‘toll like receptor 9, snp, pulmonary tuberculosis’, ‘toll like receptor 9, polymorphism, pulmonary tuberculosis’, or ‘toll like receptor 9, polymorphism, lung tuberculosis’. Only the articles in English or Chinese with an English abstract were selected. After excluding duplicates, titles and abstracts were reviewed. Studies would be selected if they: 1) were case-control studies; 2) reported genotype distribution in both cases and controls. The studies were excluded if they were: 1) review articles; 2) not related to TLR9; 3) not a human study; or 4) not related to the association between tuberculosis and host genetics. The study selection process was summarized in Figure 1. The study was approved by the Institutional Review Board of The 309th hospital of PLA.

### Data extraction

For each study, the following information was extracted from original publication: the name of first author, the year of publication, country of origin, race of study population, genotypes distribution for each polymorphism in both cases and controls, statistical characteristics of each study (sample size, gender and age distribution of cases and controls, *P* values for HWE evaluation), source of controls, and genotyping methods (Table 1).

**Table 1:** **Characteristics of the 16 studies included in the meta-analysis**

**Authors**	**Year of publication**	**Country**	**Host ethnicity**	**Age, years Mean ± SD or Mean (Range)**		**Samples n**		**Genotyping method**
				**Cases**	**Controls**	**Cases**	**Controls**	
Jahantigh, et al.	2013	Iran	Iranian population, Zahedan	51.1 ± 20	48.4 ± 14.7	124	149	PCR-RFLP
Kobayashi, et al.	2012	Indonesian	Javanese or Sundanese–Javanese	41.6 (16-92)	-	538	560	PCR-DNA chip
		Vietnam	Kinh	37.4 (15-60)	-	277	458	PCR-DNA chip
Olesen, et al.	2007	West African	West African	37.25	38.12	321	347	PCR-TaqMan SNP assays
Sa’nchez, et al.	2012	Colombia	Colombia population, Medellin	39 (26–51)	42 (25–54)	466	300	MALDI-TOF mass spectrometer
Yang, et al.	2013	China	Chinese	-	-	200	200	PCR-CHIP
Selvaraj, et al.	2010	India	south Indian population,	34.92 ± 11.42	32.33 ± 9.75	206	212	PCR-RFLP
Diana, et al.	2013	Mexico	Mexican Mazatecan	46.9 ± 17.8	42.9 ± 15.9	90	90	PCR-TaqMan assays

PCR = polymerase chain reaction; RFLP = restriction fragment length polymorphism; MALDI-TOF: chip-based matrix-assisted laser desorption⁄ionization time-of-flight.

### Statistical analyses

Hardy-Weinberg Equilibrium (HWE) was examined in controls by asymptotic Pearson’s Chi-square test for each polymorphism in each study. The association between polymorphism and TB risk was estimated with odds ratios (OR) and corresponding 95% confidence intervals (CI). Between studies heterogeneity was tested using Q test and I^2^ test, and the heterogeneity was considered significant if P-value was less than 0.05. Fixed-effects model was adopted when P-value was more than 0.05; otherwise random-effects models were used [8]. The publication bias was evaluated using Begg’s test and Egger’s test [9,10]. *P* < 0.05 was considered statistically significant. Statistical analyses were conducted with Stata 13.0 (College Station, TX).

We used ‘Meta-analysis of Observational Studies in Epidemiology’ [11] as guideline for conduction and reporting of this meta-analysis.

## Results

### Characteristics of included studies

**Figure 2:**
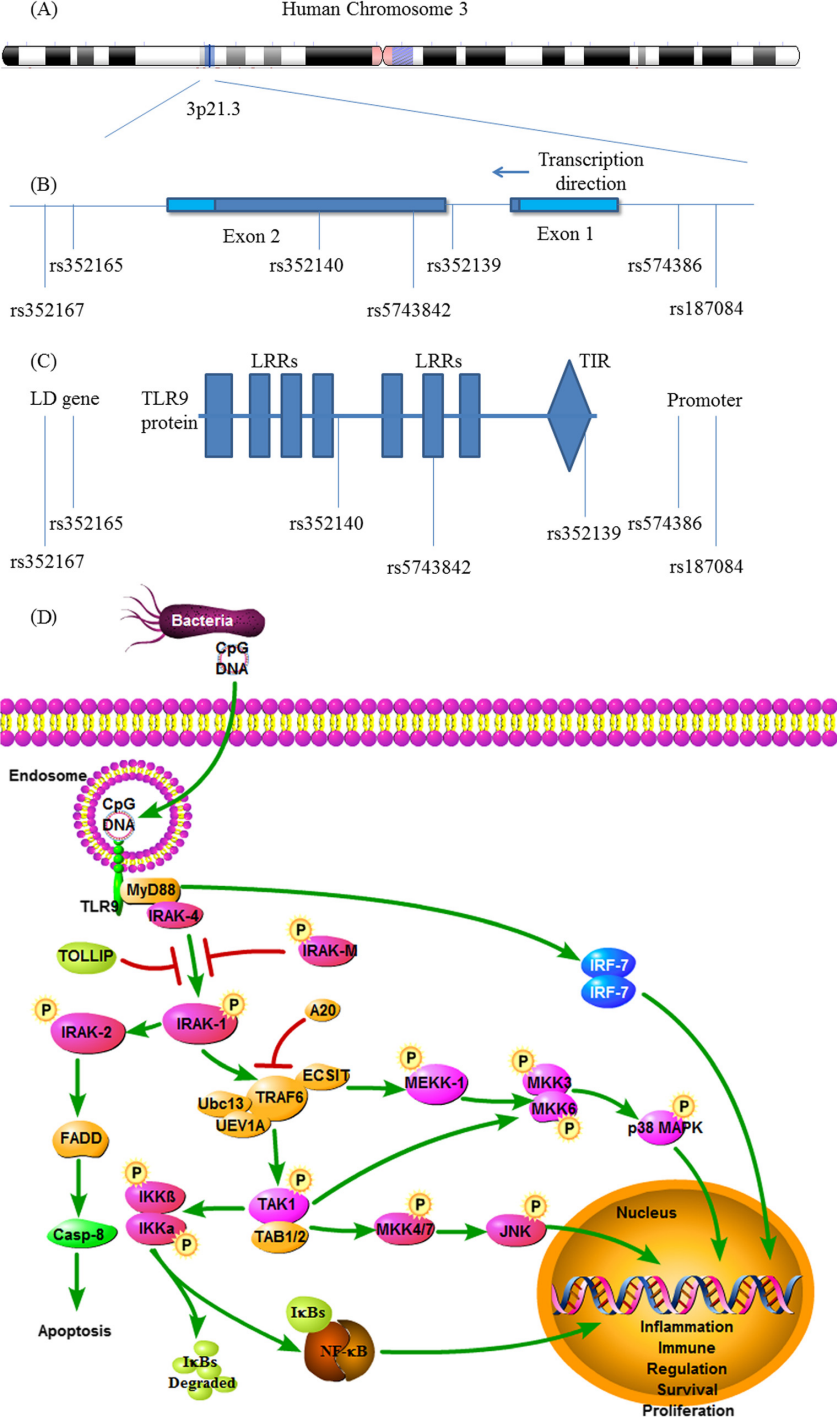
The location of TLR9 SNPs and TLR9 signaling pathway. **(A)** Schematic representation of human chromosome 3 showing location of TLR9 gene. **(B)** Schematic representation of the selected single nucleotide polymorphisms (SNPs) in TLR9. Drak regions in exon 1&2: protein coding region. **(C)** Schematic representation of selected SNPs in TLR9 protein. TLR9: Toll-Like Receptor; LRR: Leucine-rich repeat; TIR: Toll-Interleukin-1 Receptor Domain; LD: linkage disequilibrium. **(D)** The signaling pathway of TLR9. TLR9 homodimer could recruit adaptor proteins MyD88. MyD88 recruits IRAK-4 and activates NF-κB through IRAKs, TRAF6, TAK1, and IκKs, resulting in cell apoptosis or inflammatory cytokine synthesis. TLR9: Toll-Like Receptor 9; MyD88: Myeloid Differentiation Primary Response Gene 88; NF-κB: nuclear factor kappa B; IRAK: Interleukin-1 Receptor associated kinase 1; TRAF 6: TNF Receptor Associated Factor 6; TAK1: TGF-b-Actiated Kinase 1; IκK: IκB kinase; IκB: Inhibitor of NF-κB.

A total of 1745 articles were obtained from the literature search. As shown in Figure 1, after excluding the duplicates, 617 abstracts were reviewed. A total of 12 research articles that reported the association of human TLR9 gene polymorphisms and TB risk were identified. Five of them were excluded after reviewing the full-text article due to studying irrelevant polymorphism, focusing on data mining, studying the SNPs within microRNAs, or lacking the detail of SNP distribution. Finally, 8 studies from 7 articles were included in this analysis. All articles were published in English [12-18]. Overall, 7 different TLR9 SNPs were studied. Among them, there were 3 studies for rs187084 [12,14,17], 1 study for rs352165 [15], 3 studies for rs5743836 [14,17,18], 1 study for rs5743842 [18], 4 studies for rs352139 [13,16,18] (Kobayashi et al did studies on Indonesian and Vietnamese), 1 study for rs352140 [16] and 1 study for rs352167 [15]. As shown in Table 1, the race of study population ranged from African, Asian to Latin American. No studies from Caucasian were included due to lack of detailed SNP distribution. The pooled study population consisted of 4538 subjects (2222 cases and 2316 controls). The genotype and allele distributions of all the polymorphisms are shown in Table 2. In 3 studies, the genotype distributions in controls were deviated from HWE. The location of each SNP was shown in Figure 2 based on information from CNBI SNPs database.

**Table 2:** **Genotype and allele distribution of TLR-9 polymorphisms in TB and controls**

**SNP**	**Study**	**Case**					**Control**					**HWE**	
		**TT**	**TC**	**CC**	**T**	**C**	**TT**	**TC**	**CC**	**T**	**C**	**χ** ^**2**^	**P**
rs187084	P. Selvaraj. et. al.	75	91	27	241	145	84	92	32	260	156	0.66	0.42
	Jahantigh, et al.	63	51	10	177	71	82	59	8	223	75	0.39	0.53
	Olesen, et al.	171	122	25	464	172	186	132	21	504	174	0.14	0.71
rs352165	Sa’nchez, et al.	90	238	138	418	514	53	162	83	268	328	2.88	0.90
rs5743836	Olesen, et al.-a	104	154	62	362	278	101	175	66	377	307	0.40	0.53
	P. Selvaraj. et. al.	168	29	1	365	31	167	32	2	366	36	0.11	0.74
	Diana Torres-García, et. al.	82	8	0	172	8	78	12	0	168	12	0.46	0.50
rs5743842	Diana Torres-García, et. al.	0	2	88	2	178	0	0	90	0	180		
		AA	AG	GG	A	G	AA	AG	GG	A	G	χ^2^	P
rs352139	Kobayashi, et. al.(Indonesian)	199	279	59	677	397	259	233	68	751	369	1.90	0.17
	Kobayashi, et. al.(Vietnamese)	123	125	28	371	181	232	183	40	647	263	0.20	0.65
	Yang, et al.	70	89	41	229	171	68	95	33	231	161	<0.00	0.98
	Diana Torres-García, et al.	23	48	19	94	86	14	41	35	69	111	0.12	0.73
rs352140	Yang, et al.	40	88	72	168	232	32	93	71	157	235	0.03	0.87
rs352167	Sa’nchez, et al.	101	238	127	440	492	60	162	76	282	314	2.44	0.12

HWE: Hardy-Weinberg Equilibrium.

### Data synthesis by polymorphism

#### TLR9 rs187084 polymorphism

**Figure 3:**
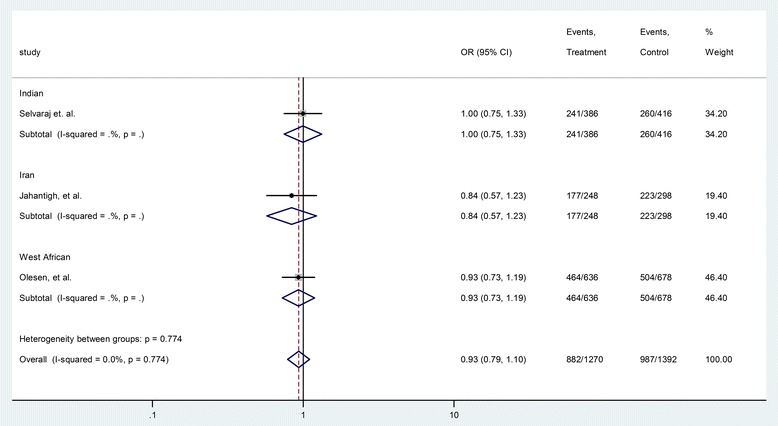
Forrest plot of the association between rs187084 and TB risk (T vs. C). Subgroup analysis was performed by ethnicity. OR: odds ratio; CI: confidence interval; df: degrees of freedom.

The Human Genome Variation Society (HGVS) name of rs187084 is NM_017442.3:c.-1486 T > C [19,20]. Three case-control studies (987cases and 405 controls) addressed the relationship between rs187084 polymorphism and the risk of TB. As shown in Table 3, the heterogeneity was not significant (P = s 0.78, I^2^ = 0.0%). The overall OR (T vs. C alleles) using fixed-effect model was 0.93 (95% CI 0.79–1.10), P =0.43. Analyses of other genetic models were also performed, and no association was identified (Table 3). The publication bias was also negligible in these genetic models (Table 3). Subgroup analysis in allele comparison (T vs. C) was performed by ethnicity in Figure 3.

**Table 3:** **Summary of different comparative meta-analysis results**

**Polymorphism**	**Genetic model**	**Participants**	**OR (95% CI)**	**Z**	**P value**	**I** ^**2**^ **%**	**P**_**het**_	**Effect model**	**Begg’s test p > │z│**	**Egger’s test p > │t│**
rs187084	TT vs. TC + CC	1331	0.93 (0.75, 1.15)	0.68	0.49	0.0	0.91	F	0.29	0.30
	CC vs. TC + TT	1331	1.12 (0.77, 1.63)	0.60	0.55	0.0	0.53	F	0.30	0.47
	TT vs. CC	784	0.85 (0.58, 1.27)	0.79	0.43	0.0	0.6	F	0.30	0.47
	TT vs. TC	1208	0.95 (0.75, 1.19)	0.50	0.63	0.0	0.91	F	0.30	0.17
	T vs. C	2660	0.93 (0.79, 1.10)	0.80	0.43	0.0	0.78	F	1.00	0.57
rs5743836	TT vs. TC + CC	1241	1.18 (0.90, 1.54)	1.18	0.24	0.0	0.82	F	0.30	0.36
	CC vs. TC + TT	1241	0.99 (0.68, 1.45)	0.06	0.95	0.0	0.58	F	1.00	_
	TT vs. CC	831	1.12 (0.72, 1.73)	0.5	0.62	0.0	0.63	F	1.00	_
	TT vs. TC	1110	1.19 (0.90, 1.57)	1.18	0.24	0.0	0.82	F	1.00	0.48
	T vs. C	2482	1.09 (0.90, 1.33)	0.89	0.37	0.0	0.72	F	0.30	0.15
rs352139	AA vs. AG + GG	2404	0.88 (0.65, 1.19)	0.83	0.40	62.1	0.049	R	0.09	0.00
	GG vs. AG + AA	2404	0.90 (0.60, 1.35)	0.51	0.61	61.9	0.049	R	0.73	0.59
	AA vs. AG	2081	0.84 (0.63, 1.12)	2.9	0.004	55.4	0.081	R	0.31	0.07
	AA vs. GG	1311	1.02 (0.65, 1.61)	0.1	0.92	62.0	0.048	R	0.73	0.24
	A vs. G	4808	0.98 (0.77, 1.25)	0.17	0.87	72.5	0.012	R	0.09	0.10

P_het_ = P value for heterogeneity; OR = odds ratio; CI = confidence interval; F = fixed-effect model; R = random-effect model.

#### TLR9 rs5743836 polymorphism

**Figure 4:**
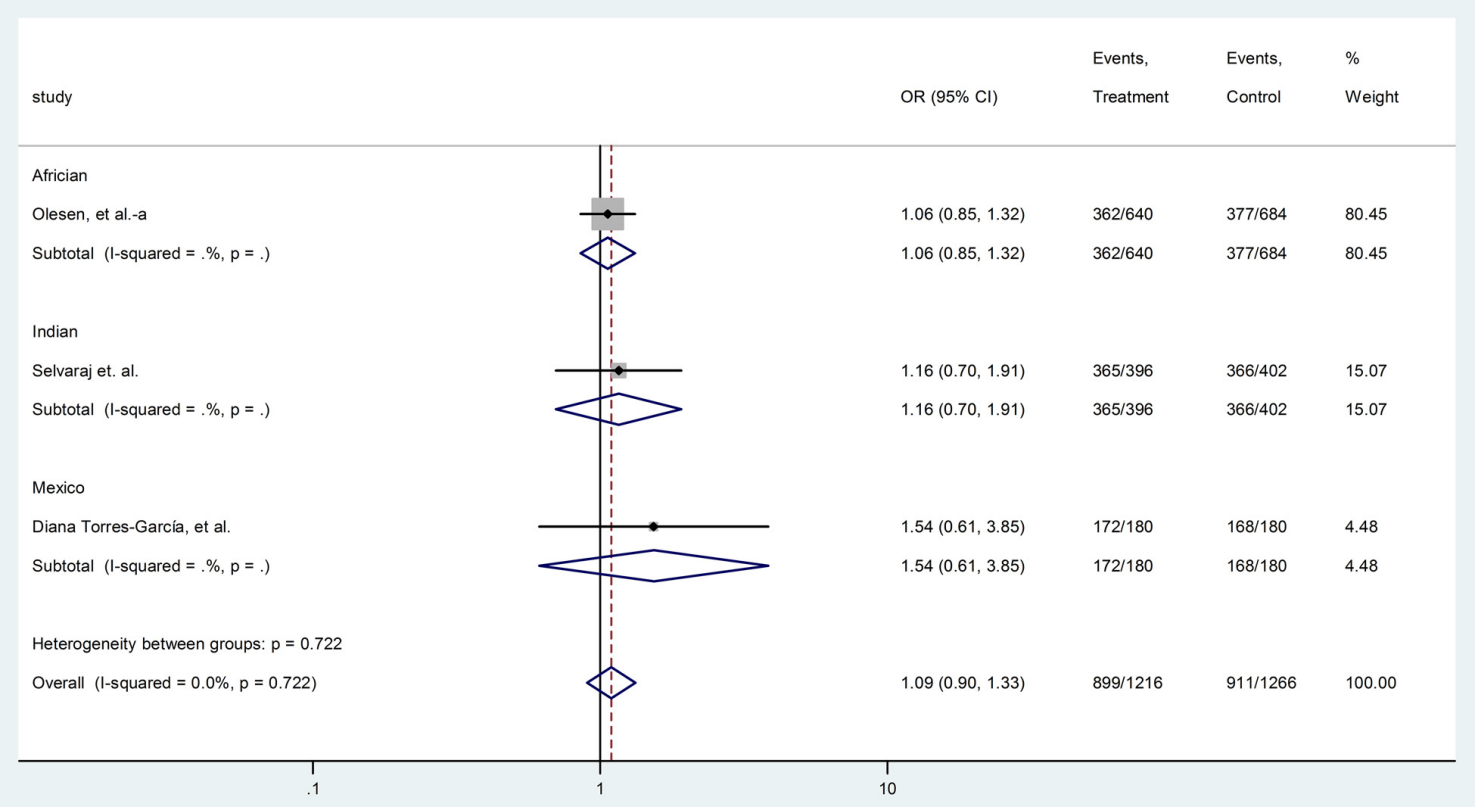
Forrest plot of the association between rs5743836 and TB risk (T vs. C). Subgroup analysis was performed by ethnicity. OR: odds ratio; CI: confidence interval; df: degrees of freedom.

The HGVS name of rs5743836 is NM_017442.3:c.-1237 T > C [19,21]. Three case-control studies (608 cases and 633 controls) were included for the analysis of the relationship between rs5743836 polymorphism and TB risk. As shown in Table 3, the heterogeneity between these studies was not significant (P = 0.72, I^2^ = 0.0%). The overall OR (T vs. C alleles) using fixed-effect model was 1.09 (95% CI 0.90–1.33), P =0.37. Analyses for other genetic models also did not identify significant association (Table 3). The publication bias was also insignificant (Table 3). Subgroup analysis in allele comparison (T vs. C) was performed by ethnicity in Figure 4.

#### TLR9 rs352139 polymorphism

**Figure 5:**
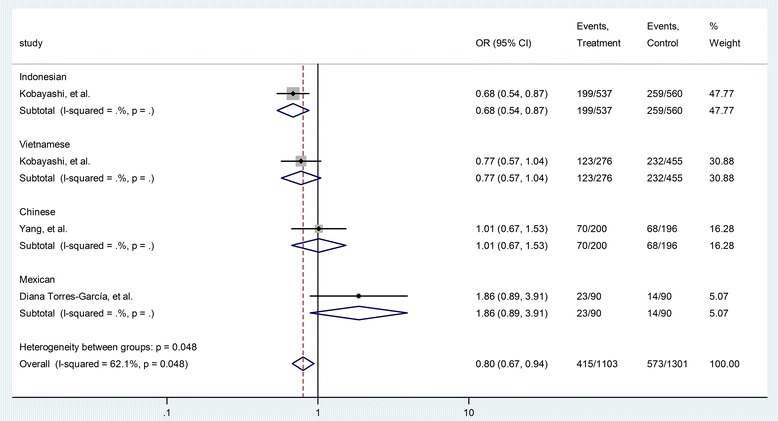
Forrest plot of the association between rs352139 and TB risk (AA vs. AG + GG). Subgroup analysis was performed by ethnicity. OR: odds ratio; CI: confidence interval; df: degrees of freedom.

**Figure 6:**
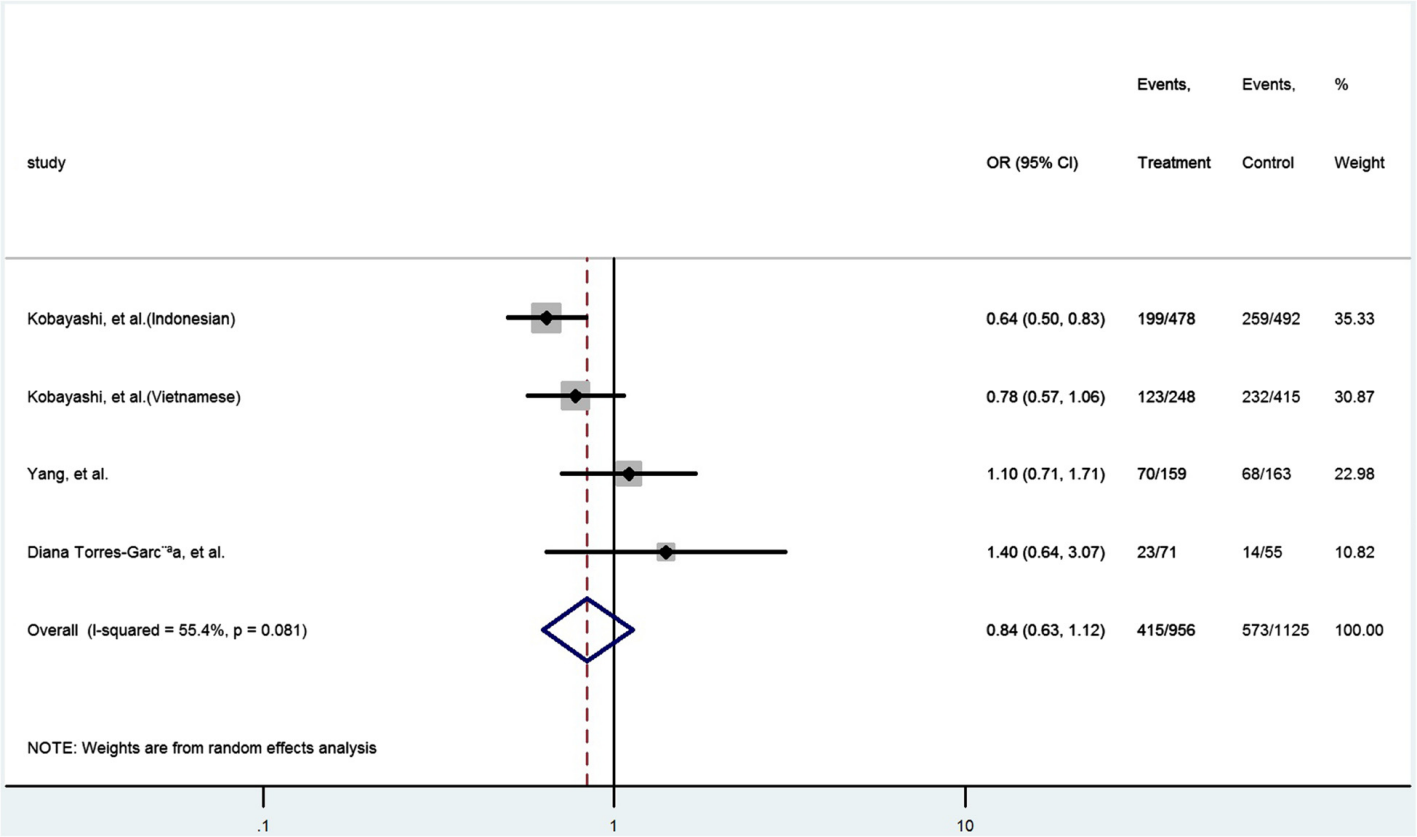
Forrest plot of the association between rs352139 and TB risk (AA vs. AG). Subgroup analysis was performed by ethnicity. OR: odds ratio; CI: confidence interval; df: degrees of freedom.

The HGVS name of rs352139 is NM_017442.3:c.4-44A > G [19,22]. Four case-control studies (1103 cases and 1301 controls) were included in the analysis of the relationship between rs352139 polymorphism and TB risk. As shown in Table 3, the heterogeneity between these studies was significant (P = 0.012, I^2^ = 72.5%). The overall OR (A vs. G alleles) using the random-effect model was 0.98 (95% CI 0.77–1.25), P =0.87. Analyses for other genetic models did not identify significant association (Table 3). Begg’s test (P = 0.09) and Egger’s test (P = 0.00) showed potential publication bias in the model of AA vs. AG + GG (Table 3). As shown in Figures 5 and 6, the Mexicans with homozygote gene AA might have higher risk TB whereas Indonesians with the same SNP genotype might have lower risk for TB. It is of note that there is only one study from each race.

#### TLR9 rs5743842, rs352165, rs352140 and rs352167polymorphism

There was only one study for each SNP, which limited further analysis. As shown in Table 4, the overall ORs for T vs. C or A vs. G were all around 1, indicating lack of association between these polymorphisms and risk of pulmonary tuberculosis. Analyses for other genetic models were also performed, and no association was found (Table 4).

**Table 4:** **Summary of different comparative SNPs results**

**Polymorphism**	**Genetic model**	**Participants**	**OR (95% CI)**	**Chi** ^**2**^	**P value**
rs5743842	TT vs. TC + CC	180	_	_	_
	CC vs. TC + TT	180	0 (0, 1.91)	_	0.50
	TT vs. CC	178	_	_	_
	TT vs. TC	2	_	_	_
	T vs C	360	_	_	_
rs352165	TT vs. TC + CC	764	1.11 (0.75, 1.64)	0.28	0.60
	CC vs. TC + TT	764	1.09 (0.78, 1.53)	0.27	0.60
	TT vs. CC	364	1.02 (0.65, 1.62)	0.01	0.92
	TT vs. TC	543	1.16 (0.77, 1.75)	0.52	0.47
	T vs C	1528	1.00 (0.81, 1.23)	0.00	0.96
rs352140	AA vs. AG + GG	396	1.28 (0.74, 2.22)	0.90	0.34
	GG vs. AG + AA	396	0.99 (0.64, 1.52)	0.00	0.96
	AA vs. AG	253	1.32 (0.74, 2.38)	0.99	0.32
	AA vs. GG	215	1.23 (0.67, 2.27)	0.52	0.47
	A vs G	792	1.08 (0.81, 1.45)	0.31	0.58
rs352167	AA vs. AG + GG	764	1.10 (0.76, 1.60)	0.26	0.61
	GG vs. AG + AA	764	1.09 (0.78, 1.55)	0.29	0.59
	AA vs. AG	561	1.15 (0.77, 1.70)	0.50	0.48
	AA vs. GG	364	1.01 (0.64, 1.58)	0.00	0.97
	A vs G	1528	1.00 (0.81, 1.23)	0.00	0.97

OR = odds ratio; CI = confidence interval.

Two individual researchers independently reviewed the studies. The inter-rater agreement, as measured by kappa statistics, was calculated with MedCalc® software [23]. The weighted kappa is 0.500; 95% CI is 0139 to 0.861, indicating a moderate agreement between 2 researchers.

## Discussion

In this study, we performed a meta-analysis to assess the association between seven extensively studied TLR9 polymorphisms (rs187084, rs352165, rs5743836, rs5743842, rs352139, rs352140 and rs352167) and TB risk. The analysis revealed an association between certain TLR9 polymorphism and TB risk. In addition, 5 different genetic models (Allele, Heterozygote, Homozygote, Dominant and Recessive model) were analyzed in all polymorphisms. A subgroup analysis by race was also performed for rs187084, rs352139 and rs5743836 polymorphisms, the studies of which included Indians, Iranian and West African, Indonesians, Vietnamese, Chinese and Mexicans. The results showed that rs187084 and rs5743836 polymorphisms were not associated with TB risk, while the association between rs352139 polymorphism and TB risk may vary by race.

We chose TLR9 gene in our analysis because it’s critical role in the incidence of TB from previous studies. Chen et al. demonstrated that cell apoptosis induced by *Mycobacterium Tuberculosis* required TLR9 in animal model [24]. Another study reported that the deficiency in a major adapter of TLR9 signaling pathway significantly increased the lethality of TB. The myeloid differentiation factor 88 knock-out mice (MyD88 −/−) died within 4 weeks after MTB infection [25]. All these evidences indicated the critical role of TLR9 in TB incidence and progression.

TLR9 is a recognition receptor in leukocytes endosome, especially in B cells, plasmacytoid dendritic cells (pDCs) and macrophages. It is localized in the endosomal and lysosomal compartments. It is one of the most important receptors for the initiation of innate immune response against intracellular pathogens. The function of TLR9 is to recognize the unmethylated Cytosine-phosphate-Guanine (CpG) sequence which usually appears in bacterial DNA [5]. In recent study, hybrid RNA-DNA chains generated by viruses during reverse transcription were recognized by TLR9 [26]. The Toll-IL-1 receptor (TIR) domain of TLR9 in intracellular space associates with myeloid differentiation factor 88 (MyD88), and becomes the initiator component of TLR9 signaling pathway. Once bacterial CpG-DNA in lysosome combines with the nucleic acid ligand of TLR9, the signaling pathway is activated. MyD88, once stimulated, recruits IL-1 receptor associated kinase 4 (IRAK-4) to TLR9 through interaction of the functional domains. Then, IRAK-1 is phosphorylated and recruits Tumor necrosis factor receptor associated factor 6 (TRAF6). Consequently the inhibitor of nuclear factor kappa-B kinase (IКK) complex is activated, leading to the activation of Mitogen-activated protein (MAP) kinases (JNK, p38 MAPK). The nuclear factor inhibiter (IкB) is then phosphorylated and the level of nuclear factor kappa-B (NF-кB) is elevated [27]. NF-кBs translocate to the nucleus, and then bind to DNA sites to regulate relevant gene transcription. Finally, inflammatory cytokines are synthesized through host defense immune response by TLRs- NF-кB pathway. The signaling pathway of TLR9 was shown in Figure 2.

SNPs rs352165 and rs352167 were located in intron 5 of aminolevulinate delta-, synthase 1 (ALAS1) gene in the downstream of TLR9. Sanchez D et al. have reported that these two SNPs were in linkage disequilibrium (LD) with rs352139 and rs352140 SNPs of TLR9 [15]. Thus they chose rs352165 and rs352167 as proxies for TLR9 gene. Lack of significant association with TB risk from our analysis indicated there was no relationship between rs352165/ rs352167 and this disease.

In our study, the association of rs352139 with TB risk varied by race. In Indonesians AA genotype is associated with lower risk of TB, while in Mexicans it is associated with higher risk of TB. The Mexicans with AA allele had 2.73 times higher risk of TB than Indonesians with the same genotype. There is no known explanation for the racial difference. Also, we observed a significant association between rs352139 polymorphism and TB risk in Indonesians for Allele, Heterozygote and Homozygote models. There was also a significant association between rs352139 polymorphism and TB risk in Mexicans for Allele, Dominant and Recessive models. Although 4 case-control studies of rs352139 were included in this analysis, there was only one study for each racial group. Therefore, our results should be interpreted with caution, and more studies with larger sample size should be considered to confirm this differential risk by race.

The SNP rs352139 is located in the intron of TLR9 gene (Figure 2). TIR domain consists of the end of exon 1, intron and a part of exon 2 which contains SNP rs352139. It is the only polymorphism located in the intron between two exons. The other polymorphisms are located in either the upstream of coding region or the exons. It is possible that the intron of TLR9 gene plays an important role in regulating the TLR-mediated immunologic response. However, it is currently unclear how this intronic SNP induces such a phenotype change. It is possible that it influences signaling by creating an alternative splicing site and thus, affecting the mRNA transcription and the final protein product. Similarly, the rs352139 SNP could be a likely marker in linkage disequilibrium with a polymorphic regulatory region that controls TLR9 expression or serve as a functional coding region SNP.

The SNP rs187084 is located in the promoter of TLR9 gene, which could create a Sp1 binding site [28]. Variant alleles of this SNP can alter the function of TLR9, impact the response to bacterial pathogens, and thereby change individual’s disease risk [29,30]. In our analysis, several studies investigated the role of rs187084 in the development of tuberculosis, but none of them showed significant association.

The SNP rs5743836 is located in the promoter region of TLR9. This SNP was thought to be associated with increased transcriptional activity and function of TLR9 [31]. In our study, rs5743836 polymorphism showed no significant association with TB risk. Nevertheless in a study of Caucasian patients, Digna Rosa Velez et al reported that the rs5743836 polymorphism had significant association with dominant and recessive models (TT vs. CT&CC, OR = 0.50, 95% CI 0.28–0.87, p = 0.015). However, we did not include these data in our analysis because only the detail of additive model was presented in the original article.

## Conclusions

This systematic review summarized the current literature on the association between TLR9 polymorphisms and TB risk. Our results indicated that rs187084, rs352165, rs5743836, rs5743842, rs352140 and rs352167 polymorphisms did not show significant association with TB risk. The rs352139 polymorphism might be associated with decreased TB risk in Indonesians whereas increased risk in Mexicans. However, the data were based on single study for each race. Whether the observed association was due to causal effect needs to be further studied.

## Abbreviations

TB: Tuberculosis
MTB: *Mycobacterium Tuberculosis*
TLRs: Toll-like receptors
PRRs: Pattern recognizing receptors
PAMPs: Pathogen-associated molecular patterns
LPS: Lipopolysaccharide
SNPs: Single nucleotide polymorphisms
CNKI: Chinese national knowledge infrastructure
HWE: Hardy-Weinberg Equilibrium
OR: Odds ratios
CI: Confidence intervals
HGVS: Human genome variation society
pDCs: Plasmacytoid dendritic cells
CpG: Cytosine-phosphate-guanine
TIR: Toll-IL-1 receptor
MyD88: Myeloid differentiation factor 88
IRAK-4: IL-1 receptor associated kinase 4
TRAF6: Tumor necrosis factor receptor associated factor 6
IКK: Inhibitor of nuclear factor kappa-B kinase
MAP: Mitogen-activated protein
IкB: Nuclear factor inhibiter
NF-кB: Nuclear factor kappa-B
ALAS1: Aminolevulinate delta-, synthase 1
LD: Linkage disequilibrium

## References

[1] Global tuberculosis report 2013 [http://apps.who.int/iris/bitstream/10665/91355/1/9789241564656_eng.pdf]

[2] Frazao JB, Errante PR, Condino-Neto A (2013). Toll-like receptors’ pathway disturbances are associated with increased susceptibility to infections in humans. Arch Immunol Ther Exp.

[3] Zhang Y, Jiang T, Yang X, Xue Y, Wang C, Liu J (2013). Toll-like receptor -1, -2, and -6 polymorphisms and pulmonary tuberculosis susceptibility: a systematic review and meta-analysis. PLoS One.

[4] Zhao S, Zhang Y, Zhang Q, Wang F, Zhang D (2014). Toll-like receptors and prostate cancer. Front Immunol.

[5] Hemmi H, Takeuchi O, Kawai T, Kaisho T, Sato S, Sanjo H (2000). A Toll-like receptor recognizes bacterial DNA. Nature.

[6] Coban C, Ishii KJ, Kawai T, Hemmi H, Sato S, Uematsu S (2005). Toll-like receptor 9 mediates innate immune activation by the malaria pigment hemozoin. J Exp Med.

[7] Tao K, Fujii M, Tsukumo S, Maekawa Y, Kishihara K, Kimoto Y (2007). Genetic variations of Toll-like receptor 9 predispose to systemic lupus erythematosus in Japanese population. Ann Rheum Dis.

[8] Kavvoura FK, Ioannidis JP (2008). Methods for meta-analysis in genetic association studies: a review of their potential and pitfalls. Hum Genet.

[9] Egger M, Davey Smith G, Schneider M, Minder C (1997). Bias in meta-analysis detected by a simple, graphical test. BMJ.

[10] Begg CB, Berlin JA (1989). Publication bias and dissemination of clinical research. J Natl Cancer Inst.

[11] Stroup DF, Berlin JA, Morton SC, Olkin I, Williamson GD, Rennie D (2000). Meta-analysis of observational studies in epidemiology: a proposal for reporting. Meta-analysis Of Observational Studies in Epidemiology (MOOSE) group. Jama.

[12] Jahantigh D, Salimi S, Alavi-Naini R, Emamdadi A, Owaysee Osquee H, Farajian Mashhadi F (2013). Association between TLR4 and TLR9 gene polymorphisms with development of pulmonary tuberculosis in Zahedan, southeastern Iran. Sci World J.

[13] Kobayashi K, Yuliwulandari R, Yanai H, Naka I, Lien LT, Hang NT (2012). Association of TLR polymorphisms with development of tuberculosis in Indonesian females. Tissue Antigens.

[14] Olesen R, Wejse C, Velez DR, Bisseye C, Sodemann M, Aaby P (2007). DC-SIGN (CD209), pentraxin 3 and vitamin D receptor gene variants associate with pulmonary tuberculosis risk in West Africans. Genes Immun.

[15] Sanchez D, Lefebvre C, Rioux J, Garcia LF, Barrera LF (2012). Evaluation of Toll-like receptor and adaptor molecule polymorphisms for susceptibility to tuberculosis in a Colombian population. Int J Immunogenet.

[16] Yang Y, Li X, Cui W, Guan L, Shen F, Xu J (2013). Potential association of pulmonary tuberculosis with genetic polymorphisms of toll-like receptor 9 and interferon-gamma in a Chinese population. BMC Infect Dis.

[17] Selvaraj P, Harishankar M, Singh B, Jawahar MS, Banurekha VV (2010). Toll-like receptor and TIRAP gene polymorphisms in pulmonary tuberculosis patients of South India. Tuberculosis (Edinb).

[18] Torres-Garcia D, Cruz-Lagunas A, Garcia-Sancho Figueroa MC, Fernandez-Plata R, Baez-Saldana R, Mendoza-Milla C (2013). Variants in toll-like receptor 9 gene influence susceptibility to tuberculosis in a Mexican population. J Transl Med.

[19] den Dunnen JT, Antonarakis SE (2000). Mutation nomenclature extensions and suggestions to describe complex mutations: a discussion. Hum Mutat.

[20] Jia X, Cong B, Zhang J, Li H, Liu W, Chang H (2014). CCK8 negatively regulates the TLR9-induced activation of human peripheral blood pDCs by targeting TRAF6 signaling. Eur J Immunol.

[21] Triantafilou K, Eryilmazlar D, Triantafilou M (2014). Herpes simplex virus 2-induced activation in vaginal cells involves Toll-like receptors 2 and 9 and DNA sensors DAI and IFI16. Am J Obstet Gynecol.

[22] Yamaguchi M, Kitagawa Y, Zhou M, Itoh M, Gotoh B (2014). An anti-interferon activity shared by paramyxovirus C proteins: inhibition of Toll-like receptor 7/9-dependent alpha interferon induction. FEBS Lett.

[23] Fleiss JLLB, Paik MC (2003). Statistical methods for rates and proportions.

[24] Chen L, Shi W, Li H, Sun X, Fan X, Lesage G (2010). Critical role of toll-like receptor 9 in morphine and Mycobacterium tuberculosis-Induced apoptosis in mice. PLoS One.

[25] Fremond CM, Yeremeev V, Nicolle DM, Jacobs M, Quesniaux VF, Ryffel B (2004). Fatal Mycobacterium tuberculosis infection despite adaptive immune response in the absence of MyD88. J Clin Invest.

[26] Rigby RE, Webb LM, Mackenzie KJ, Li Y, Leitch A, Reijns MA (2014). RNA: DNA hybrids are a novel molecular pattern sensed by TLR9. EMBO J.

[27] Blasius AL, Beutler B (2010). Intracellular toll-like receptors. Immunity.

[28] Hamann L, Glaeser C, Hamprecht A, Gross M, Gomma A, Schumann RR (2006). Toll-like receptor (TLR)-9 promotor polymorphisms and atherosclerosis. Clin Chim Acta.

[29] Roszak A, Lianeri M, Sowinska A, Jagodzinski PP (2012). Involvement of Toll-like Receptor 9 polymorphism in cervical cancer development. Mol Biol Rep.

[30] Ng MT, Van’t Hof R, Crockett JC, Hope ME, Berry S, Thomson J (2010). Increase in NF-kappaB binding affinity of the variant C allele of the toll-like receptor 9–1237 T/C polymorphism is associated with Helicobacter pylori-induced gastric disease. Infect Immun.

[31] Mollaki V, Georgiadis T, Tassidou A, Ioannou M, Daniil Z, Koutsokera A (2009). Polymorphisms and haplotypes in TLR9 and MYD88 are associated with the development of Hodgkin’s lymphoma: a candidate-gene association study. J Hum Genet.

